# Galunisertib Exerts Antifibrotic Effects on TGF-β-Induced Fibroproliferative Dermal Fibroblasts

**DOI:** 10.3390/ijms23126689

**Published:** 2022-06-15

**Authors:** Joshua M. Peterson, Jayson W. Jay, Ye Wang, Alejandro A. Joglar, Anesh Prasai, Alen Palackic, Steven E. Wolf, Amina El Ayadi

**Affiliations:** 1 Department of Pathology, University of Texas Medical Branch, Galveston, TX 77555, USA; jopeters@utmb.edu; 2 Department of Surgery, University of Texas Medical Branch, Galveston, TX 77555, USA; jwjay@utmb.edu (J.W.J.); yewang@utmb.edu (Y.W.); anprasai@utmb.edu (A.P.); alpalack@utmb.edu (A.P.); swolf@utmb.edu (S.E.W.); 3 School of Medicine, University of Texas Medical Branch, Galveston, TX 77555, USA; aajoglar@utmb.edu; 4 Division of Plastic, Aesthetic and Reconstructive Surgery, Department of Surgery, Medical University of Graz, A-8036 Graz, Austria

**Keywords:** fibrosis, TGF- β inhibition, myofibroblast, ALK5, injury

## Abstract

Dermal fibroblasts in pathological scars secrete constitutively elevated levels of TGF-β, signaling the transcription of fibrotic genes via activin-like kinase 5 (ALK5). In the present study, we examine the antifibrotic effects of galunisertib, a small-molecule inhibitor of ALK5, on fibroproliferative dermal fibroblasts in an in vitro model of wound healing. We induced fibrosis in human dermal fibroblasts with exogenous TGF-β and performed cellular proliferation assays after treatment with varying concentrations of galunisertib. Dermal fibroblast proliferation was diminished to homeostatic levels without cytotoxicity at concentrations as high as 10 μM. An in vitro scratch assay revealed that galunisertib significantly enhanced cellular migration and in vitro wound closure beginning 24 h post-injury. A gene expression analysis demonstrated a significant attenuation of fibrotic gene expression, including collagen-1a, alpha-smooth muscle actin, fibronectin, and connective tissue growth factor, with increased expression of the antifibrotic genes MMP1 and decorin. Protein synthesis assays confirmed drug activity and corroborated the transcription findings. In summary, galunisertib simultaneously exerts antifibrotic effects on dermal fibroblasts while enhancing rates of in vitro wound closure. Galunisertib has already completed phase II clinical trials for cancer therapy with minimal adverse effects and is a promising candidate for the treatment and prevention of pathological cutaneous scars.

## 1. Introduction

**Figure 1:**
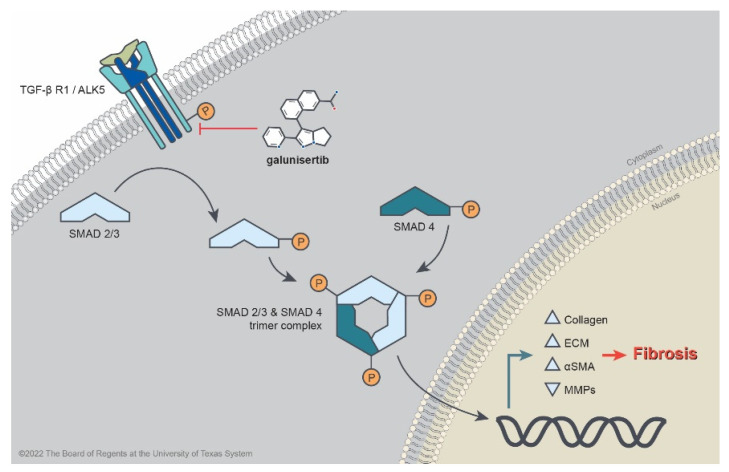
Molecular pathway depicting TGF-β-induced cutaneous fibrosis. The small-molecule inhibitor galunisertib prevents the phosphorylation of the intracellular domain on the TGF-βR-1/ALK5 serine-threonine kinase, thereby preventing downstream Smad2/3 signaling and nuclear translocation, resulting in the attenuation of fibrotic phenotypes.

TFG-β signaling pathways govern fibroproliferative phenotypes in both physiological and pathological dermal scar formation. In hypertrophic and keloid scars, the composition, quantity, and orientation of collagen fibers are altered, resulting in thick fibrous scars with a loss of compliance and elasticity [1]. The differentiation and synthetic activity of myofibroblasts during wound healing are regulated by TGF-β signaling pathways, which are disrupted in states of pathological scarring [2]. During the initial phase of physiological wound healing, TGF-β1 is released from degranulated platelets and binds to TGF-β receptor type 2, forming a heteromeric complex with TGF-β receptor type 1 (also known as activin-like-kinase 5, or ALK5), leading to the phosphorylation of its cytoplasmic kinase domain (Figure 1) [2]. Activated ALK5 recruits and phosphorylates receptor-regulated Smad proteins, Smad2 and Smad3 (Smad2/3), which then form a trimeric complex with Smad4 [3]. This complex translocates to the nucleus, ultimately inducing the transcription of extracellular matrix (ECM) genes [4]. Ultimately, a distinct TGF-β isoform (TGF-β3) that is released during the remodeling phase of wound healing attenuates ECM production by blocking TGF-β receptor activation [5]. Previous in vitro studies have shown evidence of the involvement of TGF-β in pathological scar formation: constitutively elevated levels of TGF-β1 in both hypertrophic and keloid scar fibroblasts have been reported, although the underlying cause is not known [6].

Inhibition of the TGF-β signaling pathway has been previously identified as a target for pharmacological treatment and the secondary prevention of pathological scars. Together with this canonical pathway, TGF-β binds other accessory receptors (e.g., ALK1, which stimulates Smad1/5/8 phosphorylation) to carry out diverse, essential, and variable functions in orchestrating wound healing throughout all its phases (e.g., the chemoattraction of leukocytes to the wound bed, neovascularization, the stimulation of ECM deposition, and ultimately the attenuation of ECM production) [2,7]. Numerous studies have demonstrated the in vitro and in vivo attenuation of fibrosis by targeting the TGF-β pathway with interventions such as other small-molecule inhibitors, neutralizing antibodies, and exon skipping [7,8,9,10,11,12]. For example, a small human trial employing interferon alpha 2 (which reduces the secretion of TGF-β, among other cytokines) for the attenuation of pathological scarring showed a modest benefit [13]. Such modalities have been largely experimental, and the current standard of care for the treatment of hypertrophic scars centers on scar rehabilitation and symptom management via scar revision surgery, laser therapy, corticosteroid injections, and compression therapy [14]. Keloid scarring possesses unique and incompletely understood wound healing pathophysiology, and although a consensus for optimal treatment has not been achieved, the currently employed modalities include steroid injection, botulinum toxin injection, cryosurgery, surgical excision, radiation, and local chemotherapy [15,16,17]. Clinically, topical silicone has been studied for the secondary prevention of pathological scars, although supporting evidence is poor and a meta-analysis has shown only mild-to-moderate treatment efficacy [18]. To date, no intervention directly targeting the downstream TGF-β signaling pathway has been widely implemented clinically for the treatment or secondary prevention of pathological scars.

Galunisertib is a small-molecular inhibitor of ALK5, which has not been previously studied in the context of dermal fibrosis or pathological scarring. Although other ALK5 inhibitors have previously shown effectiveness in attenuating fibrosis, galunisertib has a relatively high selectivity for the ALK5 receptor (IC50: 0.051 ± 0.005 μM), a favorable toxicity profile, and has undergone phase II clinical trials for the treatment of hepatocellular carcinoma and myelodysplastic syndrome [7,9,19,20,21,22,23]. It has also been found to be efficacious as an antifibrotic treatment in ex vivo models of liver fibrosis [24]. Given these promising properties as a potential therapeutic agent, we sought to evaluate the efficacy of galunisertib in exerting antifibrotic effects in an in vitro model of dermal wound healing and fibrosis.

## 2. Results

### 2.1. Galunisertib Normalizes Proliferation Indices in TGF-β-Induced Fibroblasts

**Figure 2:**
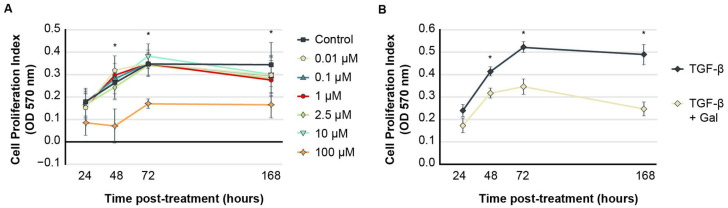
(**A**,**B**) In vitro galunisertib treatment. MTT assays show low-concentration galunisertib (0.01–10 µM) did not affect dermal fibroblast proliferation under normal culture conditions but was cytotoxic at 100 µM when compared to control (**A**), * *p* < 0.05. Treatment of FPDFs with 10 µM galunisertib significantly reduced proliferation rate after 24 h incubation (**B**), * *p* < 0.05. Data are presented as mean cellular proliferation rates ± SEM.

The treatment of human dermal fibroblasts (HDFs) induced with exogenous TGF-β to assume a fibroproliferative phenotype (FPDFs) with galunisertib at concentrations ranging from 0.01 to 10.0 µM showed similar proliferation index rates as control-treated cells between 24 and 168 h (Figure 2A). At these concentrations, proliferation indices ranged from 0.154 ± 0.102 (0.01 µM) to 0.175 ± 0.103 (0.1 µM) at 24 h and gradually increased over time. Control HDF proliferation indices were measured to be 0.177 ± 0.096 at 24 h, which increased to 0.345 ± 0.157 at 168 h. All cells treated with 0.01–10.0 µM galunisertib showed no significant differences in proliferation indices compared to vehicle-treated cells at any time point. However, at 100 µM, galunisertib significantly inhibited proliferation, substantially reduced cellular metabolic activity, and appeared to be cytotoxic to dermal fibroblasts at all examined time points compared to control cells (* *p* < 0.05). No cytotoxicity was observed in HDF controls treated with 1% DMSO.

After induction to FPDFs by TGF-β, HDF proliferation increased significantly over time relative to vehicle-treated HDFs (*p* < 0.05). The proliferation indices gradually increased from 0.241 ± 0.095 at 24 h post-treatment to 0.488 ± 0.155 at 168 h. When FPDFs were treated with 10 µM galunisertib, proliferation indices were significantly reduced compared to FPDF culture alone at all time points beyond 24 h (*p* < 0.05) (Figure 2B).

### 2.2. Galunisertib Expedites Rates of In Vitro Wound Closure in TGF-β-Induced Fibroblasts

**Figure 3:**
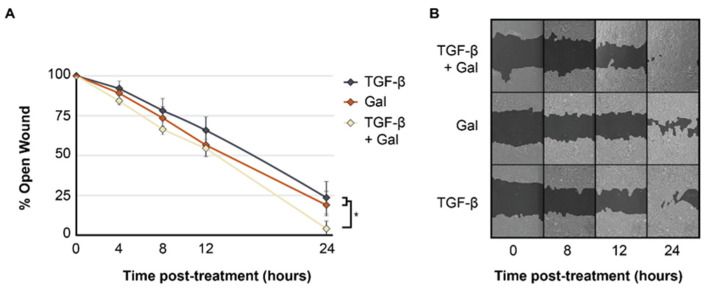
(**A**,**B**) Galunisertib increases the rate of artificial wound closure. Representative serial images of in vitro scratch assays from 0–24 h (**A**) show that galunisertib (10 µM) significantly increased the rate of in vitro wound closure of FDPFs compared to untreated FPDFs (**B**). Data are presented as mean percent open wound ± SEM (* *p* < 0.05).

The treatment of FPDFs with 10 µM galunisertib significantly increased the rate of in vitro wound closure at 24 h (4.47 ± 7.76%) compared to both untreated FPDFs (23.24 ± 16.01%, *p* < 0.05) and vehicle-treated HDFs (19.72 ± 12.06%, *p* < 0.05) (Figure 3A). However, prior to 24 h, the in vitro wound closure rates were similar among all groups. A qualitative assessment of digital images confirmed the increased migration of FPDFs treated with 10 µM galunisertib compared to HDFs treated with galunisertib or untreated FPDFs (Figure 3B).

### 2.3. Galunisertib Reduces the Expression of Fibrotic Genes and Promotes the Expression of Antifibrotic Genes in TGF-β-Induced Fibroblasts

The assessment of transcript levels by RT-q-PCR of FPDFs treated with 10 µM galunisertib for 1, 3, and 7 days (d1, d3, d7) revealed significantly reduced fold change expression compared to untreated FPDFs in COL1A1 (d1: 0.69 ± 0.06; d3: 0.48 ± 0.11; d7: 0.43 ± 0.03, *p* < 0.05), COL3A1 (d3:0.59 ± 0.12, *p* < 0.05), ACTA2 (d1: 0.84 ± 0.51; d3: 0.44 ± 0.18, *p* < 0.05), FN1 (d1: 0.94 ± 0.28; d3: 0.55 ± 0.15; d7: 0.61 ± 0.20, *p* < 0.05), and CTGF (d1: 0.07 ± 0.03; d3: 0.01 ± 0.01, *p* < 0.05) (Table 1, column: Gal + TGF-β). Additionally, a comparison of antifibrotic genes between these groups showed a significant increase in fold changes of MMP1 (d7: 11.49 ± 2.91, *p* < 0.05) and DCN (d3: 1.00 ± 0.31; d7: 1.66 ± 0.48, *p* < 0.05). Across all timepoints and genes, no detectable differences were evident between FPDFs and HDFs after treatment with galunisertib. Furthermore, no significant difference in the initial expression of any of the examined fibrosis genes was observed after one hour (d0), except in CTGF, where initial measurement of transcript levels was higher in FPDFs compared to the galunisertib-treated HDFs and FPDFs (d0: 1.55 ± 0.30 vs. 1.10 ± 0.06 and 1.07 ± 0.18, respectively; *p* < 0.05). However, the relative decrease in CTGF expression after one day of treatment in HDFs (94.7% decrease) and FPDFs (93.2% decrease) compared to untreated FPDFs (10.9% increase) was dramatic and sustained throughout all following timepoints (d3 and d7).

### 2.4. Galunisertib Diminishes the Production of Fibrotic Proteins in TGF-β-Treated Fibroblasts

**Figure 4:**
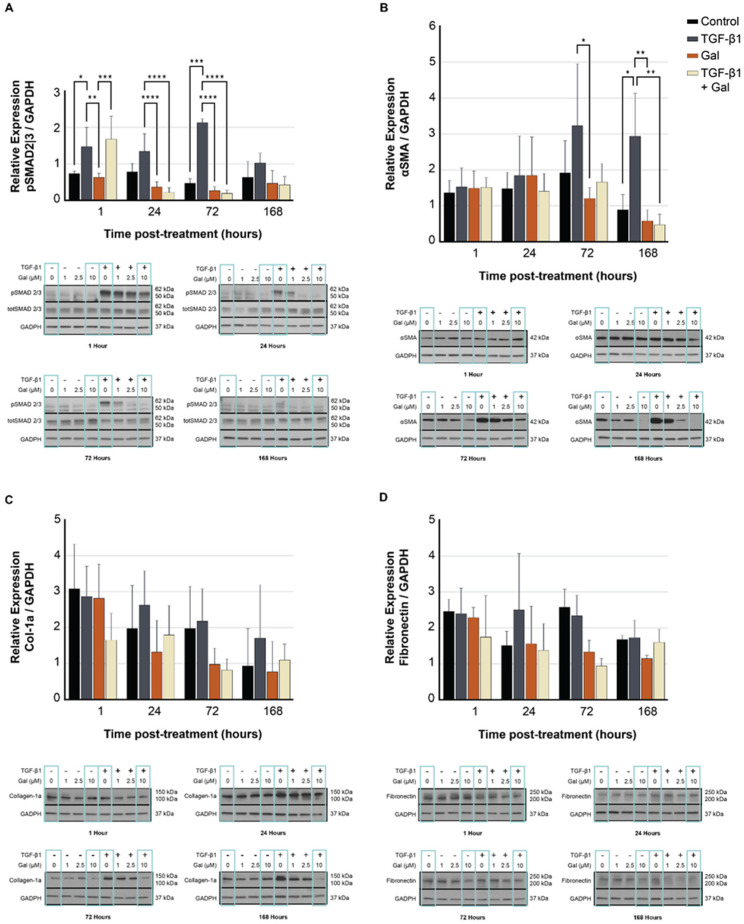
(**A**–**D**) Fibrotic protein expression of TGF-β signaling assessed by Western blot. Galunisertib (10 µM) significantly reduced Smad2/3 phosphorylation in FPDFs (**A**) up to 168 h post-treatment. Downstream αSMA protein expression (**B**) was significantly reduced after 72 h; however, collagen-1 (**C**) and fibronectin (**D**) expression were not affected at any timepoint. GAPDH served to normalize protein expression. Data are presented as mean relative expression ± SD (* *p* < 0.05, ** *p* < 0.01, *** *p* < 0.001, **** *p* < 0.0001).

Western blots confirmed decreased protein expression in FPDFs treated with galunisertib relative to untreated FPDFs in downstream targets of TGF-β signaling. Significant decreases in Smad2/3 phosphorylation (Figure 4A) were observed in treated FPDFs as soon as 1 h after the initial treatment and persisted up to 72 h post-treatment (*p* < 0.001). Beginning at 72 h post-treatment and persisting up to 168 h post treatment, αSMA expression (Figure 4B) was significantly reduced (*p* < 0.01). Similar trends were measured in collagen-1a (Figure 4C) and fibronectin (Figure 4D) at all timepoints but not at significant levels. In untreated FPDFs, Smad2/3 phosphorylation levels returned to near-baseline levels at 168 h post-induction. The FPDF protein expression of αSMA was significantly reduced with galunisertib pre-treatment at 72 h and continued through 168 h following treatment (*p* < 0.05). Similar trends were measured in collagen-1a and fibronectin at all time points but not at significant levels.

## 3. Materials and Methods

### 3.1. Reagent and Cell Preparation

Normal fibroblasts from human neonatal foreskin (referred to as human dermal fibroblasts or HDFs) were used in all experiments. HDFs were purchased from ATCC (PCS-201-010, Manassas, VA, USA). Galunisertib (LY2157299) was purchased from MedChemExpress (Monmouth Junction, NJ, USA). A stock solution of 10 mM galunisertib was prepared by dissolving in 100% DMSO. Recombinant human transforming growth factor beta-1 (rhTGF-β) was purchased from PeproTech (#100-21, Cranbury, NJ, USA). The treatment of HDFs with rhTGF-β (10 ng/mL) induced a fibroproliferative phenotype, as confirmed by the increased expression of fibrotic markers such as alpha-smooth muscle actin (αSMA) and collagen-1a. This cell population is referred to as fibroproliferative dermal fibroblasts (FPDFs).

### 3.2. MTT Cell Proliferation Assay

HDFs were cultured in 96-well plates in Dulbecco’s Modified Eagle Medium (DMEM) supplemented in 10% fetal bovine serum (FBS) in a 5% CO_2_ humidified environment until 60–75% confluent. Serial dilutions of galunisertib (0 µM, 0.01 µM, 0.1 µM, 1 µM, 2.5 µM, 10 µM, and 100 µM), together with a 1% DMSO and 5% DMSO control, were added to the FPDF and HDF cell cultures. FPDFs were incubated with galunisertib for 0, 24, 72, and 168 h without medium exchange. After incubation, 10 µL of MTT reagent (ATCC 30-1010K kit) was added to each well with a 2 h incubation time. A proprietary detergent reagent (100 µL) was added, and cells were allowed to incubate for 2 h at room temperature. The absorbance at 570 nm was then measured using a BMG LabTech Fluostar Optima spectrophotometer.

### 3.3. In Vitro Model of Wound Healing (Scratch Assay)

To assess the effect of galunisertib on in vitro wound healing, HDFs were cultured in 6-well plates in DMEM with 10% FBS and a 1% antibiotic solution until 90–100% confluent. Serum starvation was performed 24 h prior to treatment. These cells were then induced to FPDFs with rhTGF-β (10 ng/mL) and treated with either 0 µM or 10 µM galunisertib. The timing of treatment with both rhTGF-β and galunisertib varied between either 24 h before scratch (concurrent with serum starvation) or 30 min before scratch. Using a 200 µL pipette tip, wounds were created on the cell monolayer with two parallel linear scratches. Immediately following the scratch, the medium was aspirated, and cells were washed in cold PBS followed by replacement with an identical medium. Serial images of the wound area were acquired at 4 h intervals using an Invitrogen EVOS M5000 microscope. The wound size, defined as the gap between cell monolayers, was measured using TScratch software (CSElab, Swiss Federal Institute of Technology, Zürich, Switzerland).

### 3.4. Reverse Transcription-Quantitative PCR

HDFs were cultured in 12-well plates in DMEM with 2% FBS and 1% antibiotic solution. Serum starvation was performed 24 h prior to treatment. HDFs were induced to FPDFs with rhTGF-β (10 ng/mL), and both HDF and FPDF cells were treated with 0 µM or 10 µM galunisertib and allowed to incubate for 1 h, 1 day, 3 days, and 7 days. Total RNA was isolated and purified from cell lysates using an RNeasy Mini Kit (Qiagen, Germantown, MD, USA). The RNA concentration and purity were determined using the Nanodrop 3300 fluorospectrometer (Thermo Scientific, Waltham, MA, USA). RNA isolates were stored at 80 °C for less than 24 h prior to reverse transcription to cDNA using an iScript cDNA synthesis kit (BioRad, Hercules, CA, USA). Human sense and antisense primers for PCR were designed and obtained from Integrated DNA Technologies (San Diego, CA, USA) for collagen-1a (COL1A1), collagen-3a (COL3A1), fibronectin (FN1), alpha-smooth muscle actin (αSMA), connective tissue growth factor (CTGF), decorin (DCN), and matrix metalloproteinases-1 (MMP1) and -13 (MMP13). The primer pairs are listed in Table 2. Using the StepOnePlus system (Applied Bioscience, Rochester, NY, USA), a quantitative analysis of the treated HDFs was performed and the fold change of expression was calculated by the delta-delta CT method.

### 3.5. Western Blot

HDF cells were cultured in 6-well plates in DMEM with 2% FBS and a 1% antibiotic solution. Serum starvation was performed 2 h prior to treatment. HDFs were induced to FPDFs with rhTGF-β (10 ng/mL), and both HDF and FPDF cells were treated with 0 µM or 10 µM galunisertib and allowed to incubate for 1, 24, 72, and 168 h. Cells were harvested using established protocols [25], and total protein concentration was determined through a BCA assay (Thermo Fisher Scientific, Waltham, MA, USA). Electrophoresis separated 35 μg of total protein in a 4–15% gradient polyacrylamide gel under denaturing conditions and it was transferred to a PVDF membrane. The membrane was blocked in 5% bovine serum albumin for 1 h at room temperature then incubated with primary antibodies (1:1000) for the following target proteins: αSMA (Sigma cat#A5691), pSmad2/3 (Cell Signaling Technology (CST) cat#8828), Smad2/3 (CST cat#3102), collagen-1a (Abcam cat#ab34710), and fibronectin (Sigma cat# F3648). GAPDH (CST cat#2118) served as the loading control. The relative protein expression was quantified using Image J software [26].

### 3.6. Statistical Analysis

Standard parametric tests, including one-way or two-way ANOVAs with Bonferonni post hoc analyses compared the differences between the treatment groups for normally distributed data using GraphPad Prism (version 9.10, GraphPad Software, La Jolla, CA, USA). Q-Q plot assessments indicated that all datasets were normally distributed. At minimum, all experiments were performed in triplicate. Unless otherwise indicated, data are presented as means ± SD, and significance was accepted at *p* ≤ 0.05.

## 4. Conclusions

The use of exogenous TGF-β to induce the fibroproliferation of dermal fibroblasts has been previously shown to reliably mimic the effects of inflammatory wound conditions by promoting the differentiation of fibroblasts to myofibroblasts [27]. Moreover, scratch assay techniques have been widely employed as models of in vitro wound healing, serving to compare rates of cellular migration [28]. Together, these techniques serve as a basic model of the conditions that would be found during the acute inflammatory phase of dermal wound healing. As shown in the current study, galunisertib effectively suppresses the supraphysiological proliferation of dermal fibroblasts treated with TGF-β while simultaneously causing no change in the homeostatic proliferation of dermal fibroblasts at concentrations between 0.01 and 10 µM. As human phase I trials utilized galunisertib doses with peak plasma concentrations approaching 2.7 µM [29], a treatment dose of 10 µM was selected to simulate in vivo conditions by capturing peak concentrations. The potency of ALK5 inhibition by galunisertib was demonstrated by the precipitous drop in phosphorylated Smad2/3 protein levels after treatment, confirming the cellular activity of galunisertib. Furthermore, the in vitro wound closure rate of FPDFs treated with galunisertib as measured by the scratch assay exceeded that of either FPDFs alone or galunisertib-treated HDFs. The production of fibrotic proteins, including collagen-1a, αSMA, fibronectin, and CTGF, decreased, while the expression of antifibrotic transcripts, including MMP1 and decorin, increased. Taken together, galunisertib effectively exerts antifibrotic effects on dermal fibroblasts and suppresses the TGF-β-induced hyperproliferative state while not hindering homeostatic proliferation. Additionally, in the setting of cellular injury, galunisertib serves to enhance cellular migration and in vitro wound closure rates. These findings suggest that when galunisertib is employed in robust in vivo experiments it will not only diminish fibrotic scarring but may also simultaneously expedite dermal wound closure.

TGF-β plays several essential roles in the physiology of wound healing, including the chemoattraction of leukocytes to the wound bed, the stimulation of neovascularization, and the dampening of ECM production during the remodeling phase [2]. In fact, knockout models of TGF-β have demonstrated delayed wound healing, illustrating its pivotal role [30]. Galunisertib targets ALK5 downstream of TGF-β signaling, thereby hindering the expression of genes regulated by Smad2/3 while permitting other physiological effects of TGF-β. The advantage of the downstream inhibition of ALK5 is supported by our findings that not only did galunisertib avoid delaying rates of in vitro wound closure in FPDFs but, in a rather synergistic fashion, enhanced the rate of in vitro wound closure beyond what was observed in the control dermal fibroblasts. Furthermore, the observation that FPDFs treated with galunisertib experienced higher wound closure rates than HDFs treated with galunisertib demonstrates that this effect is attributable to TGF-β signaling itself and illustrates a permissive advantage of galunisertib in preserving non-fibrotic physiological effects of TGF-β by targeting a downstream mediator.

The pathophysiology of hypertrophic and keloid scars involves an imbalance in the ECM remodeling equilibrium with reduced secretion of select matrix metalloproteinases (MMPs) and increased collagen production [31]. MMP1, also known as fibroblast collagenase-1, participates in dynamic ECM remodeling by catalyzing the degradation of collagens I, II, and III. In a study assessing the effect of galunisertib on ex vivo human cirrhotic liver tissue, galunisertib was found to upregulate the expression of MMP1 transcript measured by qRT-PCR [24]. The significant upregulation of MMP1 observed in treated FPDFs reflects its capacity to shift the equilibrium of ECM remodeling towards degradation.

Pathological scars are also typified by the histological observation of disorganized collagen bundles. Decorin is a small extracellular collagen-binding protein that is necessary for the organization of ECM collagen fibrils and sequesters secreted TGF-β. It has been demonstrated to be diminished by as much as 75% in hypertrophic scars, and knockout models of decorin have shown similar patterns of collagen disorganization as those found in hypertrophic scars [32,33,34,35]. As physiological scars mature through repeated cycles of ECM remodeling, the relative levels of decorin increase over time [36]. Decorin gene expression was found to be significantly upregulated in treated FPDFs, consistent with the previously reported effects of galunisertib in other tissues [24]. The upregulation of decorin in treated FPDFs suggests that galunisertib may promote the organized deposition of ECM, as seen in physiological scarring.

The ability of galunisertib to affect ECM deposition by reducing the production of ECM collagen-1a and fibronectin was not as robust as their observed decrease in gene expression. Distinct from αSMA and pSmad2/3, both of which experienced diminished production after treatment with galunisertib, collagen-1a and fibronectin are secreted and deposited into the ECM. Moreover, unlike intracellular αSMA and pSmad2/3, the degradation of collagen-1a and fibronectin is dependent on the secretion of ECM enzymes, including MMPs. As shown here, the upregulation in MMP1 and MMP13 expression did not occur until post-treatment day 7, providing a limited time for the degradation of ECM collagens relative to controls. The background levels of ECM collagen-1a and fibronectin deposited within wells prior to treatment may also have diluted the relative apparent treatment effect. Although inconclusive, we anticipate that in vivo experiments carried out along an adequate timeframe to capture all phases of wound healing would demonstrate significant decreases in net ECM production, as the gene expression data suggest.

Previous studies demonstrated the effectiveness of galunisertib in exerting antifibrotic effects in models of both kidney fibrosis and liver fibrosis [24,37,38]. It has also been studied as an antitumor agent in hepatocellular carcinoma, pancreatic adenocarcinoma, and glioblastoma, including the completion of phase I clinical trials with no major adverse effects and sparse minor adverse effects [7,21,22,39,40]. Nonetheless, this is the first study to investigate the in vitro effects of galunisertib on dermal wound healing. While other interventions aiming to attenuate ALK5 signaling, including exon skipping, siRNA knockdown, and small-molecule inhibitors, have shown in vitro efficacy [8,12], galunisertib is particularly promising for use in vivo, owing to its relatively high selectivity for the ALK5 receptor (IC50: 0.051 ± 0.005 μM), favorable toxicity profile, and progression through phase II clinical trials [7,9,19,20,21,22,23]. Furthermore, its high hydrophobicity permits efficient translocation through the cell membrane and makes it an ideal candidate for preparation as a topical cream for localized drug delivery. Topical application would diminish its potential for severe adverse effects such as cardiac valvular ulceration, which has only been observed in animal experiments [7,40].

In summary, galunisertib demonstrates efficacy in exerting antifibrotic effects on fibroproliferative dermal fibroblasts while simultaneously enhancing the rates of in vitro wound closure. The displayed antifibrotic effects include both the suppression of fibrotic genes and a concomitant increase in the expression of antifibrotic genes. The actions of galunisertib on TGF-β-induced human dermal fibroblasts are consistent with previous results in other tissues. Overall, galunisertib exhibits promising features as a candidate drug for clinical application in disease states characterized by cutaneous fibrosis.

## Figures and Tables

**Table 1:** Fibrotic gene expression of treated human dermal fibroblasts assessed by RT-qPCR.

Gene			Treatment		
	TGF-β (Comparison Group)		Galunisertib Only		TGF-β + Galunisertib
*1 h*					
COL1A1	1.00 ± 0.04	↔	1.12 ± 0.12	↔	1.01 ± 0.02
COL3A1	1.01 ± 0.01	↔	1.30 ± 0.22	↔	1.02 ± 0.07
DCN	1.08 ± 0.14	↔	1.18 ± 0.13	↔	0.91 ± 0.00
ACTA2	1.02 ± 0.07	↔	1.04 ± 0.17	↔	0.80 ± 0.11
CTGF	1.55 ± 0.30	↓	1.10 ± 0.06 *	↓	1.07 ± 0.18 *
FN1	1.01 ± 0.06	↔	1.28 ± 0.09	↔	0.78 ± 0.20
MMP1	1.13 ± 0.16	↔	1.30 ± 0.13	↔	0.89 ± 0.02
MMP13	1.11 ± 0.12	↔	1.23 ± 0.17	↔	1.08 ± 0.24
*Day 1*					
COL1A1	1.67 ± 0.12	↓	0.57 ± 0.07 *	↓	0.69 ± 0.06 *
COL3A1	1.17 ± 0.07	↓	0.71 ± 0.17*	↔	0.77 ± 0.23
DCN	0.38 ± 0.06	↔	0.79 ± 0.11	↔	0.79 ± 0.23
ACTA2	6.33 ± 2.41	↓	0.81 ± 0.42 *	↓	0.84 ± 0.51 *
CTGF	1.72 ± 0.55	↓	0.06 ± 0.02 *	↓	0.07 ± 0.03 *
FN1	2.93 ± 1.11	↓	0.76 ± 0.15 *	↓	0.94 ± 0.28 *
MMP1	0.63 ± 0.06	↔	0.84 ± 0.17	↔	0.66 ± 0.13
MMP13	0.85 ± 0.07	↔	0.78 ± 0.05	↔	0.79 ± 0.21
*Day 3*					
COL1A1	2.02 ± 0.16	↓	0.47 ± 0.05 *	↓	0.48 ± 0.11 *
COL3A1	1.29 ± 0.17	↔	0.92 ± 0.32	↓	0.59 ± 0.12 *
DCN	0.21 ± 0.01	↑	1.39 ± 0.09 *	↑	1.00 ± 0.31 *
ACTA2	8.41 ± 2.60	↓	0.61 ± 0.27 *	↓	0.44 ± 0.18 *
CTGF	1.21 ± 0.15	↓	0.02 ± 0.01 *	↓	0.01 ± 0.01 *
FN1	4.28 ± 2.60	↓	0.69 ± 0.13 *	↓	0.55 ± 0.15 *
MMP1	0.57 ± 0.11	↔	2.90 ± 1.46	↔	1.17 ± 0.17
MMP13	0.89 ± 0.11	↔	1.31 ± 0.25	↔	1.01 ± 0.13
*Day 7*					
COL1A1	1.24 ± 0.17	↓	0.35 ± 0.01 *	↓	0.43 ± 0.03 *
COL3A1	0.86 ± 0.15	↔	1.13 ± 0.31	↔	1.18 ± 0.32
DCN	0.83 ± 0.23	↑	1.71 ± 0.55 *	↑	1.66 ± 0.48 *
ACTA2	1.69 ± 0.74	↔	0.64 ± 0.17	↔	0.63 ± 0.32
CTGF	0.31 ± 0.10	↔	No expression	↔	No expression
FN1	3.03 ± 0.88	↓	0.57 ± 0.22 *	↓	0.61 ± 0.20 *
MMP1	1.54 ± 0.38	↑	11.33 ± 3.77 *	↑	11.49 ± 2.91 *
MMP13	1.20 ± 0.11	↑	1.88 ± 0.76 *	↔	1.50 ± 0.28

Galunisertib (10 µM) significantly reduced TGF-β-induced expression of αSMA (ACTA2), collagen-1a (COL1A1), fibronectin (FN1), connective tissue growth factor (CTGF), and decorin (DCN) after 1 day of treatment. Moreover, matrix metalloproteinases-1 (MMP1) and -13 (MMP13) gene expression were significantly increased by galunisertib in the presence of rhTGF-β after 7 days of treatment, indicating that galunisertib significantly alters TGF-β-induced fibrotic signaling. Comparison to the “Galunisertib only” group showed similar results, suggestive of a rescue effect. Arrows indicate significant differences in expression relative to the comparison group (human dermal fibroblasts induced to fibroproliferative dermal fibroblasts with rhTGF-β). Data are presented as mean expression fold changes ± SD from control calculated by the ΔΔCT method; fold change = 1 reflects basal expression of the target gene (* *p* < 0.05).

**Table 2:** Primer pairs for TGF-β-induced fibroproliferative gene targets.

Target Gene	Primers (5′-3′) Sense	Antisense
18S	GGCCCTGTAATTGGAATGAGTC	CCAAGATCCAACTACGAGCTT
COL1A1	GTCACCCACCGACCAAGAACC	AAGTCCAGGCTGTCCAGGGATG
COL3A1	ATGCCCTACTGGTCCTCAGA	GGAACCAGGATGACCAGATG
DCN	CCTGATGACCGCGACTTCGAG	TTTGGCACTTTGTCCAGACCC
ACTA2	GACGAAGCACAGAGCAAAAGAG	TGGTGATGATGCCATGTTCTATCG
CTGF	CGGCTTACCGACTGGAAGAC	CGTCGGTACATACTCCACAG
FN1	GACTTCCTATGTGGTCGGAG	TGTCTTCAGCCACTGCATCC
MMP1	CTGAACGGTGATGAAGCAGCC	AGTCCAAGAGAATGGCCGAG
MMP13	CATTTGATGGGCCCTCTGGCCTGC	GTTTAGGGTTGGGGTCTTCATCTC

## Data Availability

No large datasets were generated in this study. Primary research can be obtained by contacting the authors.
